# Alterations of hand sensorimotor function and cortical motor representations over the adult lifespan

**DOI:** 10.18632/aging.102925

**Published:** 2020-03-11

**Authors:** Melina Hehl, Stephan P. Swinnen, Koen Cuypers

**Affiliations:** 1 Movement Control and Neuroplasticity Research Group, Department of Movement Sciences, Group Biomedical Sciences, KU Leuven, Heverlee, Leuven, Belgium; 2 KU Leuven, Leuven Brain Institute (LBI), Leuven, Belgium; 3 REVAL Research Institute, Hasselt University, Agoralaan, Diepenbeek, Belgium

**Keywords:** Transcranial Magnetic Stimulation (TMS), cortical motor representation, aging, intracortical inhibition/facilitation, sensorimotor performance

## Abstract

Using a cross sectional design, we aimed to identify the effect of aging on sensorimotor function and cortical motor representations of two intrinsic hand muscles, as well as the course and timing of those changes. Furthermore, the link between cortical motor representations, sensorimotor function, and intracortical inhibition and facilitation was investigated. Seventy-seven participants over the full adult lifespan were enrolled. For the first dorsal interosseus (FDI) and abductor digiti minimi (ADM) muscle, cortical motor representations, GABA_A_-mediated short-interval intracortical inhibition (SICI), and glutamate-mediated intracortical facilitation (ICF) were assessed using transcranial magnetic stimulation over the dominant primary motor cortex. Additionally, participants’ dexterity and force were measured. Linear, polynomial, and piecewise linear regression analyses were conducted to identify the course and timing of age-related differences. Our results demonstrated variation in sensorimotor function over the lifespan, with a marked decline starting around the mid-thirties. Furthermore, an age-related reduction in cortical motor representation volume and maximal MEP of the FDI, but not for ADM, was observed, occurring mainly until the mid-forties. Area of the cortical motor representation did not change with advancing age. Furthermore, cortical motor representations, sensorimotor function, and measures of intracortical inhibition and facilitation were not interrelated.

## INTRODUCTION

Healthy aging is associated with a reduction in sensorimotor function, such as for example, a higher variability of movements [1–3], slower reaction times [3–5], impaired coordination skills [6–9], and a generally lower performance level [10] in older as compared to younger adults. Commonly, manual dexterity and force decline simultaneously, potentially at the partial expense of independence as most tasks of everyday life require an efficient and dexterous handling of objects [3, 11]. These functional declines in age-related sensorimotor functions are frequently paralleled by physiological and anatomical adaptations of central structures such as the reorganization and remodeling of the brain in general and the primary motor cortex (M1) in particular [3, 12–14]. In the current study, transcranial magnetic stimulation (TMS) is applied to identify the impact of aging on the spatial reorganization within M1. TMS is a technique that allows to non-invasively study several brain function parameters. Moreover, single-pulse (SP) TMS can be used to identify a muscle’s corticospinal excitability and its spatial motor representation, while paired-pulse (PP) paradigms are used for investigating gamma-aminobutyric acid type A (GABA_A_)-ergic and glutamatergic receptor-mediated neurotransmission within M1 [15]. By administering a sufficiently strong magnetic pulse to M1, an action potential can be evoked, leading to a motor evoked potential (MEP) in the muscle(s) corresponding to the stimulated brain region [15].

Here, cortical reorganization is investigated using a TMS mapping procedure. This approach applies single TMS pulses spatially distributed over M1, with the aim to identify its spatial organization in combination with the corticospinal excitability in this area (for a review see [15, 16]). Cortical motor representations can be expressed in the dimensions of map area, reflecting the size of the surface of M1 corresponding to a motor function, map volume, defined as the sum of the mean MEP peak-to-peak amplitudes of all active points [17], and maximal MEP, the maximal value of the mean MEPs of all active points [18] (for details, see materials and methods). Thus far, TMS mapping studies investigating the effect of aging on motor representations of intrinsic hand muscles have been relatively scarce and it remains unclear how cortical reorganization, as assessed by TMS mapping, changes over the full lifespan. Previously, Coppi et al. [19] showed a decrease in cortical motor representation area of the abductor pollicis brevis (APB) in the non-dominant hemisphere in older as compared to younger adults, while the representation of the abductor digiti minimi (ADM) did not change. No alterations were demonstrated for muscle representations of the dominant hemisphere. Another study [20] reported a spatially more extensive motor representation of the first dorsal interosseus (FDI) muscle in aged adults irrespective of the investigated hemisphere which was interpreted as an indication of M1 dedifferentiation (i.e. reduced neural distinctiveness of cortical representation).

Interestingly, previous research suggested an important role of GABA_A_-related inhibition in the regulation of cortical plasticity [21]. Moreover, it was suggested that cortical plasticity is associated with reorganization of cortical representations [22]. So far, this link remains unclear. Consequently, one goal of the current study was to investigate the link between cortical motor representations and GABA_A_-ergic receptor-mediated neurotransmission of M1, using PP TMS. Additionally, the relationship between cortical motor representations and glutamatergic receptor-mediated neurotransmission, as well as the balance between glutamatergic and GABA_A_-ergic receptor-mediated neurotransmission was explored, since it is possible that an increase in facilitation [23] or an altered balance between facilitation and inhibition is linked with alterations in cortical motor representations. GABA_A_-ergic and glutamatergic receptor-mediated neurotransmission of M1 can be assessed by respectively short-interval intracortical inhibition (SICI) [24] and intracortical facilitation (ICF) [24, 25]. A meta-analysis reported contradictory evidence regarding age-related alterations in SICI and ICF [14] and pointed overall towards an absence of changes in ICF with age [14]. The same work outlined the results of 11 studies that investigated age-related SICI changes and reported a slight, but non-significant reduction in SICI in older as compared to younger adults [14]. However, this meta-analysis did not include two studies reporting a significant decrease in SICI with age [26, 27].

Evidence from TMS studies suggests that an age-related gradual loss of inhibitory modulation is associated with a decline in sensorimotor function, such as a reduction in movement speed and impaired coordination [5, 26, 28, 29]. Moreover, associations between the level of hand usage and short- and long-term corticospinal adaptations were observed. For example, studies reported a decrease in corticospinal excitability after a period of immobilization [30] or strength training [31]. Furthermore, the repeated practice of highly dexterous motor tasks over a long period of time, as seen in many musicians, has been associated with a reduced area of and overlap between different cortical representations of hand muscles [32], as well as a reduced SICI and ICF [33, 34]. However, these studies did not address the direct link between cortical representations of intrinsic hand muscles and SICI/ICF. In contrast to the above described findings observed in pianists [32], spatially more extensive motor representations were identified with advancing age and linked to slower reaction times [20]. Yet, the relationship between age-related changes of sensorimotor function, cortical motor representations, and intracortical inhibition/facilitation is still poorly understood. Regarding the full adult lifespan, only one study [26] addressed SICI and dexterity changes, revealing information about the behavioral relevance of age-related alteration in GABA_A_-ergic inhibition and the timing of those changes. More specifically, lower SICI (i.e. less inhibition) was related to a worse alternate finger tapping performance but not to solitary finger tapping, and SICI showed a gradual reduction with advancing age [26]. Further relations between changes in sensorimotor performance, motor representations, and SICI and ICF have not yet been investigated over the full adult lifespan.

Therefore, the first aim of this cross-sectional TMS study was to identify the age-related alterations of the cortical motor representations of the dominant FDI and ADM muscle, and how these changes are related to sensorimotor function (dexterity, force) over the full adult lifespan. Secondly, we investigated the course and timing of those changes. Since the FDI and ADM muscle serve different functional purposes in everyday manual tasks (respectively contributing to precision grip and power grip), the side-to-side investigation of these two muscles was deemed relevant as fine motor function might decline faster with advancing age as compared to more generic manual functions [10]. Thirdly, the link between motor representations, sensorimotor function and intracortical inhibition and facilitation was explored as inhibition and facilitation potentially contribute to lifespan changes in sensorimotor function and changes in M1 representation.

We hypothesized (1) an age-related decline in sensorimotor function, (2) an overall increase in TMS-derived cortical motor representations, and (3) age-related changes in cortical motor representations being more pronounced for the FDI than for the ADM muscle. Furthermore, we expected a negative relationship between sensorimotor function and an age-related increase in cortical representation area and volume (4). Finally, we expected an age-related decline in sensorimotor function (5a) and increased cortical representation area and volume (5b) to be linked with reduced intracortical inhibition and increased facilitation.

## RESULTS

Prior to the main analysis, data was checked for covariation. No significant relationships were identified between age and resting motor threshold (rMT) or age and the score on the Baecke Questionnaire of Habitual Physical Activity. Moreover, neither did the rMT show an association with the cortical motor representation measures area (AREA) and volume (VOL)or the maximal MEP (MAXMEP) of the FDI and ADM muscles, nor was the cortical motor representation area associated with the head size of the participant. Furthermore, the level of physical activity did not have an effect on measures of cortical excitability (rMT and maximal MEP) or cortical motor representation area and volume (all R²_adjusted_ < 0.04, all p > 0.05).

Prior to MEP analysis, background activity was checked. Of all TMS trials, < 1% trials with a high background electromyographic (EMG) activity (root mean square (RMS) > 20 μV) was discarded from further analysis.

Detailed model statistics of all regression models given below can be found in Supplementary Table 1.

### Effect of aging on sensorimotor performance

**Figure 1:**
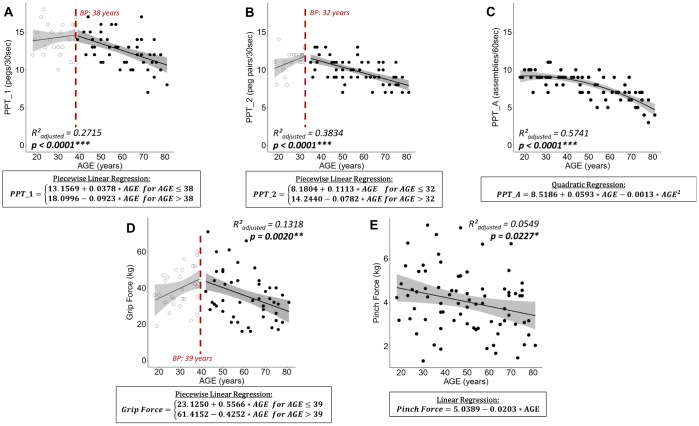
**Plots of best fitting regression models for changes in sensorimotor performance over the lifespan.** For piecewise linear regressions the breakpoint (BP) is indicated. (**A**) Purdue Pegboard Test unimanual peg placement with the dominant hand (PPT_1), (**B**) Purdue Pegboard Test peg placement with both hands (PPT_2), (**C**) Purdue Pegboard Test assembly task (PPT_A), (**D**) maximal Grip Force, and (**E**) maximal Pinch Force. For piecewise linear regressions, data points below and above the breakpoint are represented by open and filled points, respectively. Below each plot the best fitting regression model is stated in a rectangle. Ribbons depict the 95% confidence interval of the fit. Significant p-values are indicated with asterisks (*** p < 0.001; ** p < 0.01; * p < 0.05) and printed in bold.

Data of all participants (n = 77) were included in the analysis. Regression analysis revealed a relationship between age and performance on the Purdue Pegboard Test. Age-related changes on the unimanual subtest of the Purdue Pegboard Test (PPT_1) were best fitted by a piecewise linear regression (R²_adjusted_ = 0.2715, p < 0.0001, Figure 1A) with an increase of performance until the age of 38 years and a subsequent performance decrease. Changes over the lifespan on the two-handed peg placement task (PPT_2) revealed a similar pattern and were best fitted by a piecewise linear regression (R²_adjusted_ = 0.3834, p < 0.0001, Figure 1B) with a breakpoint at 32 years. The age-related decline in performance in the assembly task of the Purdue Pegboard Test (PPT_A) was best regressed by a quadratic function (R²_adjusted_ = 0.5741, p < 0.0001, Figure 1C).

Analyzing the changes in force over the lifespan, grip force followed a piecewise linear relationship with an increase in grip force until the age of 39 and a subsequent decrease in force (R²_adjusted_ = 0.1318, p = 0.0020, Figure 1D). Pinch force was best modelled by a linear regression (R²_adjusted_ = 0.0549, p = 0.0227, Figure 1E) with a constant decline in pinch force with advancing age and a high inter-subject variability.

All models fulfilled the assumptions of homoskedasticity and normal distribution of residuals.

### Effect of aging on cortical motor representations

Mapping of the cortical motor representation of the dominant hemisphere could not be performed in one participant as almost no MEPs could be evoked at 80% of the maximum stimulator output, being the maximally permitted intensity to stimulate as stated in the ethical approval. Therefore, 76 observations were included in the analysis for the FDI cortical motor representation. In another nine participants mapping of the ADM muscle at 115% rMT of the FDI elicited no MEPs in the ADM. Thus, 67 observations were included in the analysis for the ADM cortical motor representation.

**Figure 2:**
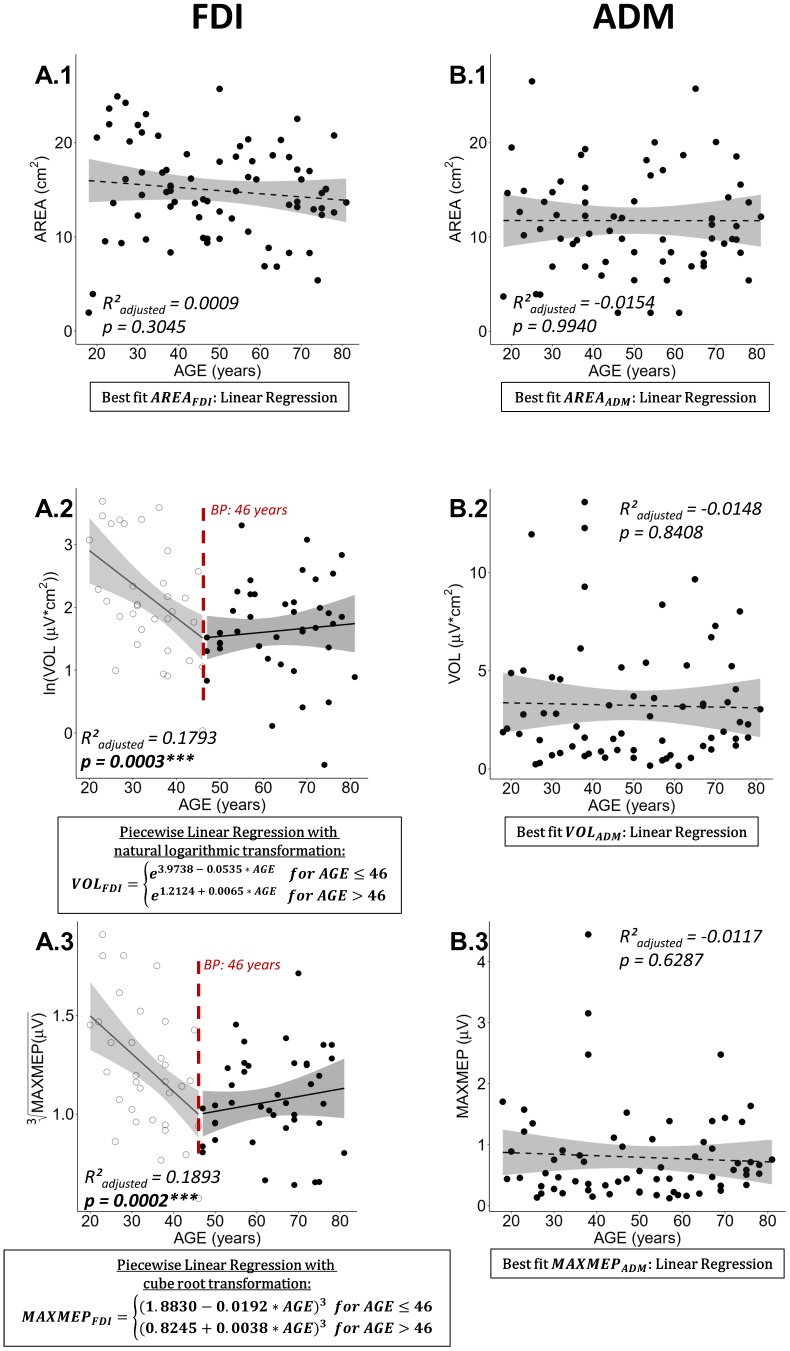
**Best regression fits for cortical motor map parameters area (AREA), volume (VOL), and maximal motor evoked potential (MAXMEP) of first dorsal interosseus (FDI) and abductor digiti minimi (ADM) muscle.** Estimates of significant regression models are stated in a rectangle below the plot and the regression is indicated in the plot by a solid line. For non-significant relationships, the best fit, on which the R²adjusted and p-value are based, is stated; a dashed line represents a non-significant regression. Ribbons depict the 95% confidence interval of the fit. Significant p-values are indicated with asterisks (*** p < 0.001; ** p < 0.01; * p < 0.05) and printed in bold.

Neither AREA for FDI, nor any of the mapping parameters for ADM showed a significant relationship with age (see Figure 2A.1 and 2B.1–3; all R²_adjusted_ < 0.0009, all p > 0.3045). Nevertheless, after natural logarithmic and cube root data transformation respectively and removal of two influential data points to fulfill model assumptions, the age-related differences of VOL and MAXMEP for the FDI were modeled optimally by a piecewise linear regression (Figure 2A.2: ln(VOL) with R²_adjusted_ = 0.1793, p = 0.0003; Figure 2A.3: ³√(MAXMEP) with R²_adjusted_ = 0.1893, p = 0.0002). Both models show a decrease until the breakpoint of 46 years and a subsequent stabilization. Detailed information on the process of regression modelling can be found in Supplementary Figure 1.

**Figure 3:**
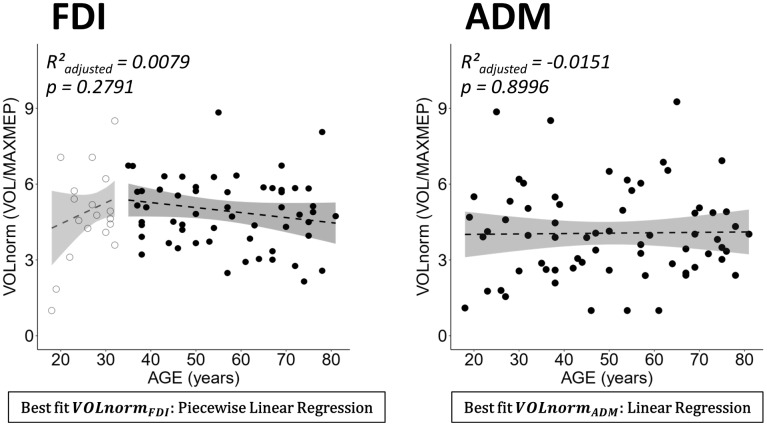
**Normalized volume (VOLnorm = volume (VOL)/maximal motor evoked potential (MAXMEP)) for dominant first dorsal interosseus (FDI) (left panel) and abductor digiti minimi (ADM) (right panel) by age.** Below each plot the best fitting model is stated in a rectangle. Ribbons depict the 95% confidence interval of the fit. Both regressions were non-significant.

Due to the similar pattern of age-related differences in VOL and MAXMEP, a post-hoc correlation analysis has been conducted. VOL and MAXMEP of the FDI showed a strong positive association (Spearman’s ρ = 0.9421, p < 0.0001). All correlations between AREA, VOL, and MAXMEP are reported in Table 1. In an additional post-hoc analysis, VOL was normalized to MAXMEP amplitude and no age-related changes in normalized VOL were observed (Figure 3).

**Table 1:** Spearman’s rank correlations coefficients (ρ) between area (AREA), volume (VOL), and maximal motor evoked potential (MAXMEP) for first dorsal interosseus (FDI) muscle (grey cells) and abductor digiti minimi (ADM) muscle (black cells).

				**ADM**	
**Spearman's ρ**	**p-value**	**Spearman's ρ**	**p-value**	**Spearman's ρ**	**p-value**
**AREA**		0.8087	<0.0001***	0.5052	<0.0001***
0.6300	<0.0001***	**VOL**		0.8843	<0.0001***
0.4298	0.0001***	0.9421	<0.0001***	**MAXMEP**	
Spearman's ρ	p-value	Spearman's ρ	p-value	Spearman's ρ	p-value
**FDI**					

Significant p-values are indicated with asterisks (*** p < 0.001; ** p < 0.01; * p < 0.05) and printed in bold.

### Link between sensorimotor performance and cortical motor representations

For the same reasons as explained above (see Effect of aging on cortical motor representations), 76 and 67 observations were included in the analysis for FDI and ADM, respectively. For the FDI, there were no significant relationships between measures of cortical motor representations (AREA, VOL) and measures of motor performance (PPT_1, Grip Force, Pinch Force) (all p > 0.05). Likewise, no significant relationships between measures of cortical motor representations (AREA, VOL) and measures of motor performance (Grip Force) could be identified for the ADM (all p > 0.05).

### Link between sensorimotor performance, cortical motor representations and resting-state intracortical inhibition/facilitation

For SICI and ICF, data of one subject’s ADM was excluded from further analysis as more than 50% of the trials had to be removed for at least one condition (SP, PP 3 ms, or PP 10 ms) due to high background EMG activity. As described for the motor maps, data of one further subject is missing due to difficulties to elicit a sufficient number of MEPs in the FDI.

No significant relationships between measures of inhibition or facilitation (SICI and ICF respectively) and measures of motor performance (PPT_1, Grip Force, Pinch Force) could be identified (all p > 0.05). Moreover, there were no relationships between measures of inhibition or facilitation (SICI and ICF respectively) and measures of cortical motor representation (AREA, VOL) (all p > 0.05). Furthermore, our data did not reveal any significant differences in SICI and ICF over the lifespan (see Supplementary Figure 2).

## DISCUSSION

The present cross-sectional study provides three main findings. Firstly, older adults exhibiting a typical age-related decline in sensorimotor function showed a reduction in cortical motor representation volume and maximal MEP amplitude of the FDI muscle as compared to younger adults, with changes occurring mainly until the mid-forties followed by a subsequent stabilization. These alterations with age were not present in the ADM muscle. Secondly, the decline in sensorimotor function could not be linked to changes in cortical motor representations. Finally, measures of resting-state intracortical inhibition and facilitation were not associated with changes in sensorimotor function and cortical motor representations.

### Effect of aging on sensorimotor performance

The observed age-related decline in sensorimotor function is in line with hypothesis 1, in which we predicted a decline in sensorimotor function with advancing age. This finding was supported by other studies that reported an age-related decline in grip [35–38] and pinch force [11, 37, 39], as well as Purdue Pegboard Test performance [11, 40–44].

Our data revealed that grip force peaked at the age of 39. Studies that aimed to identify normative values for grip force yielded comparable maximal force [35–38] and a curvilinear relationship with a peak at about 40 years of age [36–38]. While our data revealed a negative linear relationship between pinch force and age, other work that analyzed the effect of age on pinch force in clusters of five years identified a curvilinear relation between lateral pinch force and age, resulting in slightly higher values, peaking between the age of 35 and 44 in men and between 55 and 59 in women [37] and subsequently declining into older age [39]. These differences in maximal force and the course of its relationship with age (linear vs. curvilinear) can partially be explained by differences in hand posture during measurement. For example, in the current study the palmar pinch has been investigated, while normative values related to the lateral pinch [35, 37] (for grasp definitions see Feix et al. [45]). Nevertheless, previous work applying a pinch force measurement similar to the one used in the current study, demonstrated comparable results. Specifically, a decline of approximately 20% in pinch force between a group of young (mean: 27.7 years) and older (mean: 70.5 years) adults and a more pronounced decline in grip as compared to pinch force has been demonstrated [11].

Dexterity measures for the unimanual subtest and assembly subtest of the Purdue Pegboard Test showed a rather comparable course with advancing age, whereas for the bimanual subtest a slightly different pattern was identified. More specifically, for the unimanual and the assembly subtest, performance was relatively stable until the mid-thirties and subsequently declined into older age. For the bimanual task, there was first an increase in performance until the age of 32, followed by a decline lasting into older age. Possibly the low performance of some younger individuals on this task resulted in overfitting of the regression model, although the model assumptions were met. Nonetheless, our results are comparable to normative data clustered into five-year divisions, showing peak performance at 26 to 30 years for the unimanual and at 21 to 25 years for the bimanual peg task, while for the assembly task the youngest group, aged 15 to 20 years, performed best [40]. Further in line with our results, normative data shows a relatively constant age-related decline in performance across all three tasks for adults aged 40 years and above, analyzed in age clusters of ten years [42, 43].

Overall, our sensorimotor performance results are comparable to normative data and therefore provide a benchmark for a valid interpretation of the neurophysiological data as well as for investigating the link between neurophysiology and sensorimotor performance.

### Effect of aging on cortical motor representations

The present study revealed a decline in volume and maximal MEP value of the dominant hemisphere until the age of 46, followed by a stabilization into old age. This result was only found for the FDI, whereas no age-related changes in cortical motor representation were identified for the ADM. No alterations in area of motor representations were found, neither for the FDI nor for the ADM. Hence, these findings are only supporting the latter part of our hypotheses, in which we predicted an overall increase in TMS-derived cortical motor representations (hypothesis 2) and that these age-related changes would be more pronounced for the FDI than for the ADM (hypothesis 3). The similar pattern of reduction in volume and maximal MEP can be explained by their high interdependence, since volume is defined as the sum of MEP amplitudes of active points. Especially the maximal MEP amplitude and cortical motor representation volume of the FDI showed a strong positive association (see Table 1).

In contrast to our results, which revealed no changes in area of the dominant FDI and ADM cortical motor representations, other mapping studies identified alterations of small hand muscle motor representations with advancing age when comparing groups of young and older adults. For example, Bernard and Seidler [20] reported a spatially more extensive motor representation of the FDI muscle in aged adults irrespective of the investigated hemisphere. On the contrary, Coppi et al. [19] compared the spatial extent of APB and ADM cortical motor representations of young and older adults and found a decline of the representation area only for the non-dominant APB, but not for the dominant APB or both ADM muscles. Therefore, our findings reporting no changes in cortical motor representation area of the FDI and ADM muscle, are only partially in line with previous findings [19, 20] that revealed no alterations of the motor representation area of the ADM with increasing age, while for muscles involved in fine manipulations such as the FDI and APB contradictory findings were reported. It is likely that differences in findings reported above can be explained by various factors, such as: methodological differences in the mapping protocol, reliability of the TMS measurements (number of pulses, use of a neuronavigational system, etc.) and differences in the definition/calculation of cortical motor representation.

The volume of the cortical motor representation of the FDI decreased with advancing age. Interestingly, when normalizing volume to the maximal MEP amplitude [46], no age-related changes were observed (see Figure 3). This result points towards age-related changes in excitability rather than cortical reorganization as underlying mechanism for the observed decrease in volume. Regarding the maximal MEP amplitude, our findings revealed a decrease with advancing age. This finding is in line with other studies that reported a smaller MEP amplitude in the FDI of a group of older as compared to younger adults for the resting FDI at 120% [47] and 130% rMT [48]. In contrast with our findings, other studies using intensities ranging from 110% to 150% rMT [28, 49–52] reported no age-related changes in the mean MEP amplitude between groups of young and older adults.

There are several findings that can help to unravel the mechanism underlying the observed age-related decrease in MEP amplitude. For doing so, we must keep in mind that TMS-derived MEPs are a result of cortical, spinal and peripheral pathway cooperation. Firstly, there is evidence for age-related deterioration at the peripheral level. With advancing age, there is a decline of the maximum compound muscle action potential (CMAP, also: M-wave) amplitude [53–55], which is defined as the action potential of a skeletal muscle elicited by a supramaximal electrical stimulation of its corresponding efferent nerve [56]. It is suggested that this age-related decrease in CMAP results from increased desynchronization of motor unit activation, a decreased proportion of fast-twitch fibers and a generally lower contractile speed of the muscle fibers [54]. Secondly, other studies report evidence for age-related changes in MEP amplitudes at the corticospinal level. In this respect, Pitcher et al. [18] reported that a group of older as compared to younger adults had equal maximal MEP amplitudes when taking peripheral changes into account by normalizing the MEP to the CMAP. Nevertheless, older adults required higher TMS stimulation intensities relative to the maximal stimulator output to reach 50% and 100% of their maximal MEP [18], which can be interpreted as an age-related decrease in corticospinal excitability. This finding is consistent with the current findings, as submaximal stimulation intensities were used to acquire the maximal MEP amplitude. Interestingly, the age-related decrease in corticospinal excitability in the current study was not paralleled with changes in rMT (see Supplementary Figure 3). With respect to the effect of aging on rMT, the literature shows contradictory results. On the one hand, there is evidence for an age-related increase in rMT ([50, 57, 58], for meta-analysis see Bhandari et al. [14]), while on the other hand and in line with the current findings, a number of studies reported no changes in rMT when comparing groups of young and older adults [18, 28, 48, 52, 55, 59–62].

### Link between sensorimotor performance and cortical motor representations

Although we hypothesized a negative relationship between motor performance and metrics of cortical motor representations (hypothesis 4), no links between sensorimotor performance (dexterity and force) and cortical motor representations (area, volume and maximal MEP) were identified. This finding is partially in line with the literature. Similar to our results, Coppi et al. [19] found no correlations between the cortical motor representations of APB and ADM (area and maximal MEP) in young and older adults and measures of dexterity and force (nine-hole peg test, finger-tapping and grip force). Sale and Semmler [55] investigated the link between measures of corticospinal excitability and hand dexterity (Purdue Pegboard Test and single/alternate tapping task) and force (index finger abduction) in groups of young and older adults. While older adults showed a declined performance in all functional measurements, only the alternate tapping task and force measure were weak but positively associated with the MEP, without substantial differences between young and older adults. Nevertheless, it should be noted that the results and conclusions of these studies were based on much smaller sample sizes (respectively n = 31 and n = 20, grouped into young and older adults [19, 55]) as compared to the current sample (n = 77, continuously distributed over lifespan).

Although links between cortical motor representations and sensorimotor performance were absent in the current study, several motor learning and training studies, however, did demonstrate a link. For example, dexterity training [63] and strength training [31] have been associated with respectively an increase or decrease of corticospinal excitability, while immobilization led to a decrease in excitability [63] and a longer immobilization duration correlated with a decrease in map area [64]. Interestingly, microstimulation studies in non-human primates identified a systematic increase in cortical motor representations, associated with repetitive execution of finger movements, but only when the task included a motor learning component [65]. In contrast, no changes in motor maps were reported when executing a simple repetitive motor task without a motor learning component [66], suggesting a vital role of motor learning for the reorganization of cortical motor representations. Therefore, a possible explanation for the absence of a link between cortical motor representations and sensorimotor performance in the current study could be the absence of a systematic motor learning paradigm in the normal aging process.

### Link between sensorimotor performance and resting-state intracortical inhibition/facilitation

In contrast with hypothesis 5a, predicting a link between declined sensorimotor function and altered intracortical inhibition and facilitation, no relationships between sensorimotor performance measures and SICI/ICF measurements were identified in the present work. In line with the present results, Marneweck et al. [67] reported no link between age-related differences in SICI and dexterity (Purdue Pegboard Test and force matching task) comparing a group of young and older adults. Nevertheless, the same study reported an association between atypical facilitation during SICI measurements (i.e. facilitation instead of the expected inhibition) and decreased Purdue Pegboard Test performance [67]. Moreover, Heise et al. [26] showed that more resting-state SICI (i.e. more inhibition) was related to a better alternate finger tapping performance, but not to solitary finger tapping. Additionally, SICI at rest and task-related SICI modulation were strongly correlated and both reduced with advancing age, analyzing age as a continuous variable [26].

The absence of a link between sensorimotor performance and measures of inhibition/facilitation in the current study was accompanied by the lack of age-related changes in SICI, ICF, and the ratio between ICF and SICI (see Supplementary Figure 2). This finding is interesting, as evidence on age-related changes of ICF is equivocal. Whereas some studies indicated less facilitation in older adults [27, 58], others found no age-related changes [59–61, 68, 69]. Results for SICI are even more contradictory since some studies yielded more inhibition [58, 62, 69], less inhibition [26, 27, 67], or no SICI changes with advancing age [28, 48, 52, 59–61, 68, 70, 71]. Likely, small sample sizes and differences in TMS protocols account for these conflicting results, as well as the fact that only two studies [26, 60] investigated age as a continuous variable.

### Link between cortical motor representations and resting-state facilitation/ inhibition

The present study also investigated the link between cortical motor representations and measures of inhibition and facilitation (hypothesis 5b), as previous studies suggested a close relationship between cortical plasticity and intracortical inhibition [72–75], as well as between plasticity and reorganization of cortical representations [22]. The importance of disinhibition for cortical plasticity and motor learning in humans has been suggested by Ziemann et al. [21]. In their work, a motor learning task generated stronger cortical and sensorimotor performance changes during ischemic nerve block-induced disinhibition, while an increase in GABA_A_-ergic receptor-mediated inhibition, induced by lorazepam administration, prevented those changes. Nevertheless, the present study yielded no relationship between cortical motor representations and SICI/ICF measurements. To the best of our knowledge, so far, no other studies investigated the link between age-related changes in cortical motor representations and intracortical inhibition/facilitation.

### Limitations

Firstly, hotspot and rMT were determined for the FDI and then used for the mapping and SICI/ICF measurement of FDI and ADM. Consequently, this might lead to a suboptimal targeting of the ADM, possibly resulting in slightly different outcome measurements as compared to the targeting of both muscles separately. Nonetheless, this approach is common practice [17, 63, 76, 77], especially when researchers are interested in more than one intrinsic hand muscle at the same time. Furthermore, this procedure substantially reduces experiment duration and there is evidence that PP TMS is relatively insensitive to targeting several muscles simultaneously [60, 63].

Secondly, it should be acknowledged that the contribution of the ADM in daily activities is less specific as compared to the FDI and therefore its contribution to hand function is more difficult to capture. In the present study grip force, a common activity of daily living, has been favored over isolated pinky abduction force. The rationale for this approach was to link cortical motor representations to daily life tasks. Probably, the contribution of the ADM to the grip force measurement is relatively limited and therefore, the link between grip force and the neurophysiological data of the ADM is less straightforward.

Lastly, in the present study, a cross-sectional design has been used. While this is a common and highly feasible approach, it poses a general limitation as systematic generation effects, such as environmental influences, cannot be excluded. A longitudinal or accelerated longitudinal design would be more appropriate to control for these effects. However, most laboratories do not have the resources to carry out these challenging research designs. Moreover, longitudinal studies are also inherent to limitations such as restricted generalizability, dealing with missing data, etc.

## CONCLUSIONS

We demonstrated an age-related reduction in cortical motor representation volume of the FDI, mainly occurring until the mid-forties, in the absence of changes in cortical motor representation area. Moreover, a strong link between cortical motor representation volume and maximal MEP amplitude was observed, suggesting that volumetric reduction was mainly driven by a decline in corticospinal excitability. No such changes were observed for the ADM. Furthermore, cortical motor representations, sensorimotor function, and measures of intracortical inhibition and facilitation were not related to each other. We observed changes in sensorimotor function over the lifespan with a marked decline starting around the mid-thirties.

## MATERIALS AND METHODS

### Participants

In this cross-sectional lifespan study 77 healthy volunteers over the full adult lifespan (age range 18-81 years, mean age ± SD: 49.38 ± 18.00, 36 female, 4 left-handed) were included. At the start of the study, participants completed the Edinburgh Handedness Inventory [78] (mean absolute lateralization quotient ± SD: 91.54 ± 15.55), the Montreal Cognitive Assessment (MoCA) [79] (mean score ± SD: 28.23 ± 1.65, range 24-30), and the Baecke Questionnaire of Habitual Physical Activity (self-reported) [80, 81] (mean score ± SD: 8.25 ± 1.12; final scores can range from 3 – least physically active, to 15 – most physically active). An overview of the participant characteristics can be found in Table 2. Recruitment took place in Flanders, Belgium on community and university level. Prior to inclusion, subjects were screened and were excluded from participation if they reported any central nervous system diseases, psychiatric disorders, medication affecting the central nervous system, history of brain surgery or injury, or presence of contraindications for TMS [82]. Participants gave full written informed consent prior to study participation according to the latest amendment of the Declaration of Helsinki [83]. The study protocol was approved by the local ethics committee (University Hospital Leuven; reference S62231).

**Table 2:** Participant characteristics.

**Category**	**Total**	**18-30 years**	**31-40 years**	**41-50 years**	**51-60 years**	**61-70 years**	**71-81 years**
**Participants (%)**	77 (100%)	14 (18.2%)	15 (19.5%)	13 (16.9%)	10 (13.0%)	13 (16.9%)	12 (15.6%)
**Age (years)**	49.38±18.00	24.43 ± 3.84	35.40 ± 3.09	46.69 ± 2.75	55.50 ± 2.51	66.31 ± 3.01	75.42 ± 2.64
**Female (%)**	36 (46.8%)	7 (50.0%)	6 (40.0%)	7 (53.8%)	5 (50.0%)	7 (53.8%)	4 (33.3%)
**Left-handed**	4	2	1	1	0	0	0
**|EHI LQ|**	91.54 ± 15.55	76.50 ± 19.49	93.13 ± 9.10	96.03 ± 9.97	96.64 ± 7.11	98.60 ± 5.04	90.30 ± 23.22
**MoCA**	28.23 ± 1.65	29.14 ± 1.41	28.33 ± 1.50	28.00 ± 1.68	28.20 ± 1.87	28.38 ± 1.50	27.17 ± 1.70
**Baecke**	8.33 ± 1.14	8.54 ± 0.89	8.31 ± 1.65	8.67 ± 0.99	8.09 ± 1.05	8.48 ± 1.25	7.80 ± 0.54

Pooled group data and data for subdivisions of six age groups are reported for illustrative purposes. Data is displayed as total number or as mean ± standard deviation.

Abbreviations: |EHI LQ| = absolute value of the lateralization quotient assessed by Edinburgh Handedness Inventory; MoCA = Montreal Cognitive Assessment; Baecke = Baecke Questionnaire of Habitual Physical Activity.

### Electromyographic recordings (EMG)

EMG signals for the FDI and ADM muscle, contralateral to the stimulated hemisphere, were collected using surface Ag-electrodes (Bagnoli™ DE-2.1 EMG Sensors, DELSYS Inc, Boston, MA, USA) fixed onto the prepared skin (3M™ Red Dot™ Trace Prep 2236, 3M Health Care, St. Paul, MN, USA) over the belly of each muscle with single-use double-sided adhesive skin interfaces (DELSYS Inc, Boston, MA, USA). The reference electrode was placed on the bony parts of the dorsal wrist. Raw EMG signals were collected (Bagnoli-4 EMG System, DELSYS Inc, Boston, MA, USA), filtered for 50 Hz noise (HumBug, Quest Scientific, North Vancouver, BC, Canada), amplified (gain = 1000), bandpass filtered (20-2000 Hz), digitized at 5000 Hz (CED 1401 micro, CED Limited, Cambridge, UK), and stored on a computer for offline analysis.

### Transcranial magnetic stimulation (TMS)

TMS was applied using a 70 mm figure-of-eight coil (MC-B70, outer coil winding diameter 2 x 97 mm, 150° angled) connected to a MagPro X100 stimulator (MagVenture A/S, Farum, Denmark) to deliver biphasic SP and PP TMS to M1 of the dominant hemisphere. To ensure accurate placement and orientation of the coil throughout the entire experiment, optically tracked neuronavigation was used (Brainsight®2, Rogue Research Inc, Montreal, Quebec, Canada). The coil handle was pointed backwards, 45° away from the midline and the coil center was positioned tangentially to the scalp [84, 85]. During TMS, participants were seated in a chair with their forearm pronated. EMG signals of the FDI and ADM were continuously monitored, and participants were encouraged to relax in order to keep the RMS of the EMG signal below 5 μV in between TMS pulses.

**Figure 4:**
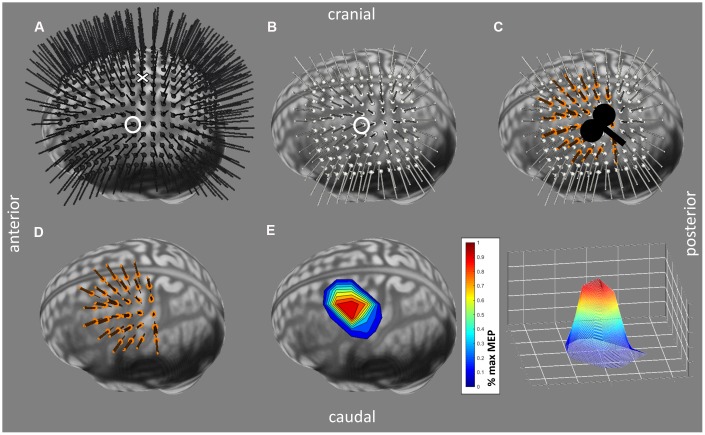
**Mapping Procedure performed with the Brainsight®2 software (Rogue Research Inc, Montreal, Quebec, Canada).** (**A**) Grid used for searching the hotspot of 19x19, 1 cm-spaced predefined target locations and their trajectories around the vertex, visualized in black. The target representing the vertex is indicated by a cross, the hotspot by a circle. (**B**) Standardized mapping grid (11x11, 1 cm-spaced) around hotspot (circled target). Predefined target locations and trajectories are visualized in white. (**C**) Stimulated targets and their trajectories (in orange) are visualized on top of the mapping grid. Stimulation was performed in a 45° angle. (**D**) Similar to (**C**) without the mapping grid. (**E**) Cortical motor representation processed in Matlab with averaged motor evoked potential (MEP) values per point normalized to the individual maximal MEP.

For mapping the cortical representation, a standardized procedure was performed (see Figure 4). Firstly, head size was measured from right to left pre-auricular point and from nasion to inion (highest point of the external occipital protuberance as palpated); then the vertex was determined as the intersection of those two lines (according to the EEG 10-20 system [86]). Secondly, using the Brainsight® software, a 1 cm-spaced rectangular 19x19 grid of targets centered around the vertex was projected over the full scalp to search for the hotspot of the FDI (see Figure 4A), defined as the scalp location with the strongest and most consistent MEP in the FDI averaged over 5 consecutive TMS pulses. Thirdly, the rMT at the hotspot was defined as the lowest intensity resulting in at least 5 out of 10 MEPs larger than 50 μV peak-to-peak amplitude in the relaxed FDI [87]. Finally, the motor representations of the FDI and ADM were mapped using an intensity of 115% rMT [19, 76] and administering 8 consecutive pulses (inter-trial interval: 3 s ± 20%) per target point, starting at the hotspot. Subsequently, points located on a 1 cm-spaced 11x11 grid centered around the hotspot were targeted in turn, proceeding spirally clockwise until all active points (defined as points with at least 4 out of 8 MEPs ≥ 100 μV peak-to-peak amplitude in at least one of the target muscles) were surrounded by inactive neighbor points (see Figure 4B–4E).

Following TMS mapping, SICI and ICF were assessed at the hotspot of the FDI, to measure GABA_A_-ergic and glutamatergic receptor-mediated neurotransmission respectively. The conditioning stimulus (CS) was set at 80% rMT [24] and the test stimulus (TS) was set to an intensity that elicited unconditioned MEPs of ≈ 1 mV peak-to-peak amplitude. For SICI, the interstimulus interval (ISI) was set at 3 ms [24], for ICF at 10 ms [24, 25]. Forty-five trials (15 SP and 15 PP for each ISI in a semi-randomized order) were administered. During the TMS mapping and the SICI/ICF measurement all participants were blinded from EMG signals and watched a slideshow of landscape pictures to promote a stable level of excitation.

### Sensorimotor performance

Grip force and palmar pinch force were measured using respectively a hydraulic hand dynamometer (Model SH5001, Saehan Corporation, Masan, Korea) and a pinch force sensor (LCM302-200N, Omega Engineering Inc, Norwalk, CT, USA). For both force measurements, participants were standing upright with the upper arm in neutral position, the lower arm in 90° elbow flexion and the wrist in mid-position between supination and pronation. They were verbally encouraged to perform optimally during three consecutive trials and the maximally generated force was analyzed.

Manual dexterity was administered with the Purdue Pegboard Test (Model 32020, Lafayette Instrument Company Inc, IN, USA). The test consists of 4 subtests that were each administered once per participant: peg placement with the dominant hand, peg placement with the non-dominant hand, peg placement with both hands simultaneously, and an assembly task, where constructions consisted of a peg, two washers and a collar. For the unimanual and bimanual peg placement subtest, respectively, the number of pegs or pairs placed within a 30-second period were recorded. For the assembly subtest, the number of completed assemblies in 60 seconds was documented.

### Data processing

**Figure 5:**
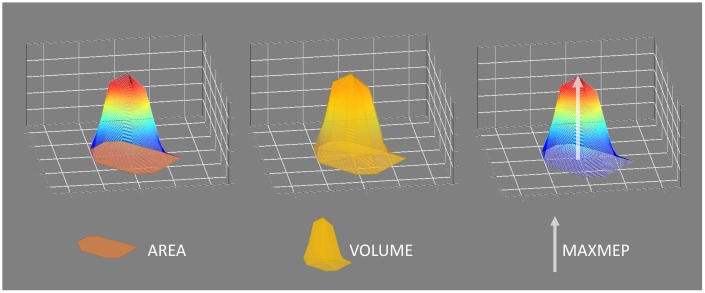
**Visualization of the cortical motor representation parameters: area, volume, and maximal motor evoked potential (MAXMEP).** Area was measured in cm² by calculating the area of a polygon expanding over all active points. Volume was measured in μV*cm² and defined as the sum of the mean motor evoked potential (MEP) peak-to-peak amplitudes of all active points, serving as an approximation of the three-dimensional integration of the map. The maximal mean MEP value of all active points was obtained as MAXMEP and measured in μV.

Cortical motor representations were assessed for the FDI and ADM and were expressed as map area (AREA) and map volume (VOL), both computed using MATLAB® (R2018b, The MathWorks Inc, Natick, MA, USA). AREA was measured by calculating the area of a polygon expanding over all active points (i.e. points that elicited at least 4 out of 8 MEPs with a peak-to-peak amplitude ≥ 100 μV). VOL was defined as the sum of the mean MEP peak-to-peak amplitudes of all active points: $VOL=\sum_{i=1}^{j}(\bar{MEP}(i)\times 1\;cm^{2}\text{|}\;i=all\;active\;scalp\;locations\text{)}$ serving as an approximation of the three-dimensional integration of the map. Furthermore, the maximal mean MEP among all active points was obtained (MAXMEP) (for visualization see Figure 5).

SICI and ICF were expressed as a ratio of the mean PP MEP amplitude over the mean SP MEP amplitude (mean MEP_PP_/mean MEP_SP_), where values < 1 indicate inhibition, while values > 1 indicate disinhibition/ facilitation. Furthermore, the ratio between both measures (ratio ICF/SICI) was calculated to express the balance between facilitation (ICF) and inhibition (SICI).

For all TMS procedures, trials with an EMG RMS exceeding 20 μV in the period 100-50 ms prior to the TMS pulse, or in case of a PP trial to the CS, were excluded from analysis.

Head size was approximated by calculating an elliptic surface with half the distance nasion-inion as radius *r*_1_ and half the distance right to left preauricular point as radius *r*_2_: *head size* = *r*_1_ * *r*_2_ * *π*.

### Statistical analysis

Statistical analyses were performed using R (Version 3.5.1, R Core Team 2018 [88]; *locfit* package version 1.5-9.1 [89]; *ggplot2* package [90] for visualizations) with α set to 0.05.

In a first step, a locally weighted regression method (local linear fit, tri-cube weight function, smoothness (alpha) = 0.5, as proposed by Cleveland [91]; no robustness) was performed for visual inspection of the shape of the data. Locations of breakpoints in the fit were identified based on the locally weighted regression estimates.

**Figure 6:**
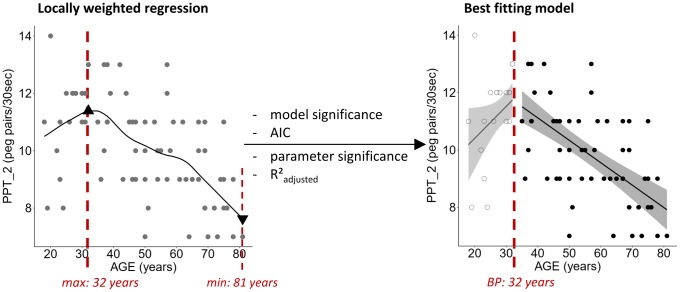
**Exemplary demonstration of locally weighted regression fit for the two-handed Purdue Pegboard Test (PPT_2) including the position of the calculated maximal and minimal value of the fit (triangles).** The optimal fitting model, here a piecewise linear regression with the breakpoint (BP) at 32 years, was chosen based on significance of the model and its parameters, R²_adjusted_, and Akaike Information Criterion (AIC). Ribbons depict the 95% confidence interval of the fit.

In a second step, linear, polynomial (until 4^th^ order), and piecewise regression models (based on the identified breakpoint; Figure 6) were calculated. Piecewise linear regression was performed based on the following regression model with a dummy variable *D* that distinguishes between x-values above and below the defined breakpoint (*X_BP_*):

$$\begin{array}{c} \hat{Y}=\beta_{0}+\beta_{1}X+\\ \beta_{2}(X-X_{BP})\times D\;\;with\;\;D=\{\begin{array}{c} 0\;for\;X\leq X_{BP}\\ 1\;for\;X>X_{BP} \end{array} \end{array}$$

$$\begin{array}{c} \hat{Y}=\beta_{0}+\beta_{1}X+\\ \beta_{2}(X-X_{BP})\times D\;\;with\;\;D=\{\begin{array}{c} 0\;for\;X\leq X_{BP}\\ 1\;for\;X>X_{BP} \end{array} \end{array}$$

In case *X* ≤ *X_BP_*, the parameter *β*_2_ exerts no effect on the regression, whereas for *X* > *X_BP_* the intercept and the slope are influenced by *β*_2_, as it can be seen in the transformation of the initial equation. Significant models were then compared based on the lowest Akaike Information Criterion (AIC). Furthermore, significance of the parameter estimates for slope and the R²_adjusted_ value were investigated. The best fitting regression based on all criteria was identified.

In a last step, the model assumptions (homoscedasticity and normal distribution of the residuals) were checked by visual inspection of the normalized residuals histogram, the quantile-quantile (Q-Q) plot of the normalized residuals, and the Cook’s distance plot of the residuals. In case assumptions were not met, data transformation and/or analysis of influential data points, as identified by the Cook’s distance plot, was performed.

In order to serve the purpose of identifying the timing and course of lifespan changes, this approach using linear, polynomial, and piecewise linear regression models has been favored over other approaches such as the comparison of different age groups or a correlation analysis. While these approaches might be more commonly used, categorizing continuous data leads to a loss of information, compromising its statistical power and leading to a higher risk of false positive results [92].

## Supplementary Material

Supplementary Figures

Supplementary Table 1
