# Supplementary material for: Alterations of hand sensorimotor function and cortical motor representations over the adult lifespan
Source: Aging (Albany NY). 2020 Mar 11;12(5):4617–40. doi: 10.18632/aging.102925 (PMC7093194; doi:10.18632/aging.102925)
Supplement: Supplementary Figures [file aging-12-102925-s002..pdf]

SUPPLEMENTARY FIGURES

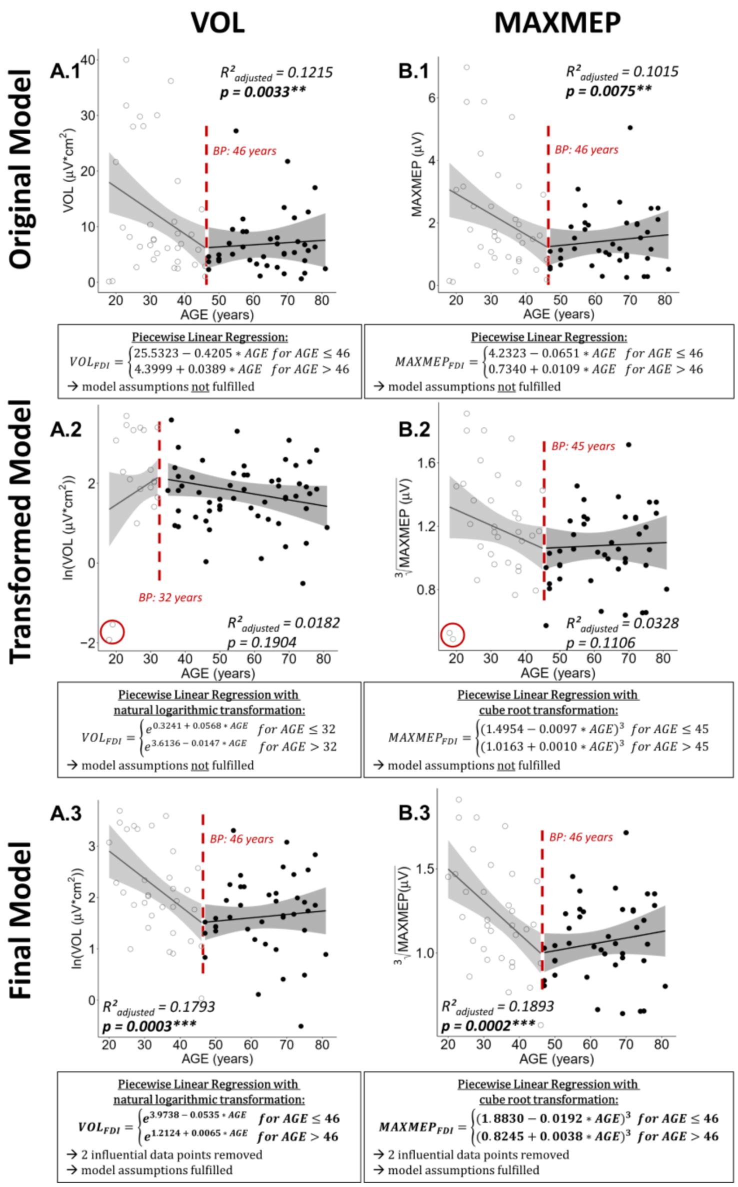

Supplementary Figure 1. Best fitting regression models for age-related differences in volume (VOL) and maximal motor evoked potential (MAXMEP) of the first dorsal interosseus (FDI) cortical motor representation. The age-related differences of VOL and MAXMEP for the FDI were modeled best by a piecewise linear regression, both with a decrease until the breakpoint at 46 years, followed by a relative stabilization (original model: see **A.1** and **B.1** for VOL and MAXMEP, respectively). As initially the model assumptions were violated (mainly due to heavy tails of the residual distribution), y-variables were transformed to satisfy the assumptions of homoskedasticity and normal distribution of the residuals: (**A.2**) regression of natural logarithmic transformation of VOL by AGE, (**B.2**) regression of cube root transformed MAXMEP by AGE. However, this led to an increased influence of influential data points (as identified by Cook's distance plots; influential data points encircled in panels **A.2** and **B.2**) and therefore a shift of the breakpoint and a substantial decline in fit. The influence of influential data points on the model was analyzed by stepwise removing them and remodeling the regression. After removal of two influential data points (representing the same participants for VOL and MAXMEP, aged 18 and 19 years old), assumptions of homoskedasticity and normal distribution of the residuals were fulfilled, resulting in the final models (VOL: **A.3**; MAXMEP: **B.3**). Both final models show a decrease until the breakpoint of 46 years and a subsequent stabilization. Model estimates are given in a rectangle below each plot. Ribbons depict the 95% confidence interval of the fit. Significant p-values are indicated with asterisks (*** $p < 0.001$; ** $p < 0.01$; * $p < 0.05$) and printed in bold.

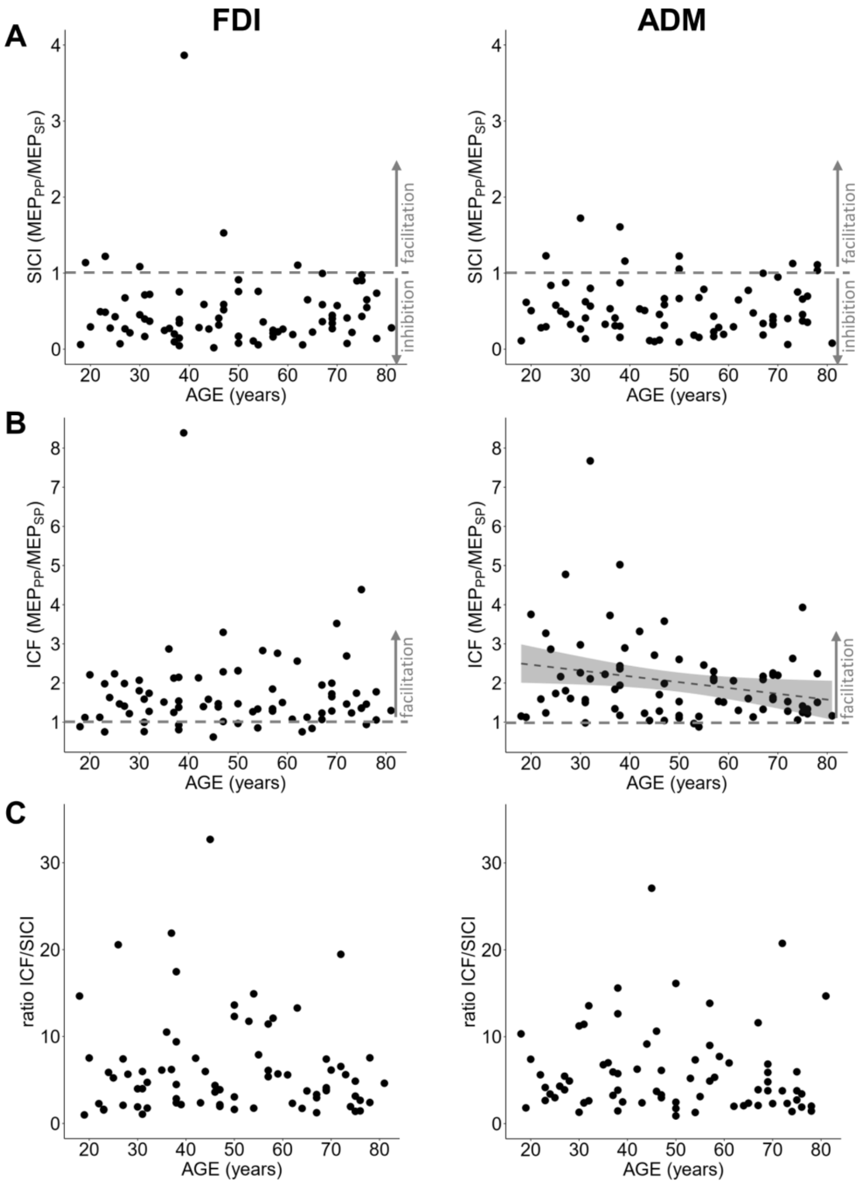

Supplementary Figure 2. Relationships of short-interval intracortical inhibition (SICI) and intracortical facilitation (ICF) with age. Left and right column show for first dorsal interosseus (FDI) and abductor digiti minimi (ADM) respectively (**A**) SICI by age, (**B**) ICF by age, and (**C**) ratio ICF/SICI by age. SICI and ICF are depicted as a ratio of the unconditioned stimulus (motor-evoked potential (MEP) of the paired pulse (PP) over MEP of the single pulse (SP)), with values > 1 implying facilitation and values < 1 indicating inhibition. A linear relationship between age and ICF for the ADM has been found ($p = 0.034$, $R^2_{adjusted} = 0.047$, right panel row (**B**) dashed line with ribbon representing the 95% confidence interval of the fit) that did not fulfill model assumptions. All approaches to comply with those (transformation, removal of influential data points) led to non-significant results (all $p > 0.05$, all $R^2_{adjusted} < 0.032$). No other relationships of ICF, SICI and ratio ICF/SICI with age (all $p > 0.05$, all $R^2_{adjusted} < 0.015$) have been identified.

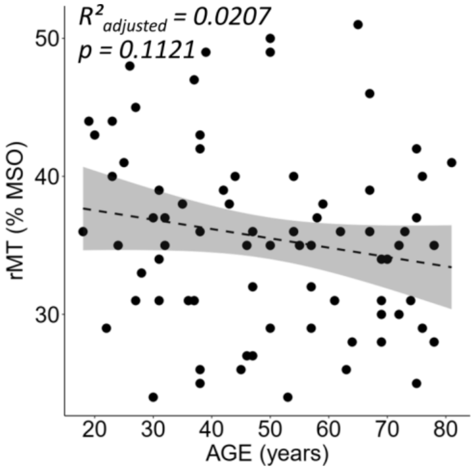

Supplementary Figure 3. Resting motor threshold (rMT) in % of the maximal stimulator output (MSO) by age. Fitting a linear regression (dashed line with 95% confidence interval of the fit) resulted in the best model. Nevertheless, no significant relationship of age and rMT has been identified.
