# Supplementary material for: Alterations of hand sensorimotor function and cortical motor representations over the adult lifespan
Source: Aging (Albany NY). 2020 Mar 11;12(5):4617–40. doi: 10.18632/aging.102925 (PMC7093194; doi:10.18632/aging.102925)
Supplement: Supplementary Table 1 [file aging-12-102925-s001..pdf]

SUPPLEMENTARY TABLE

Supplementary Table 1. Point estimates, standard error (SE) and statistics for regression models with AGE as independent variable.

Dependent variable<br>(best fitting regression model)		Estimate:	SE:	t-value:	P<br>(>|t|):		R^2adjusted:	F-statistic<br>(DF NUM; DF DEN):	P(>F):
PPT_1 ~ AGE	Intercept	13.1569	1.5716	8.372	<0.0001	***	0.2715	F (2;74) = 15.16	<0.0001***
(piecewise linear<br>regression,	β1	0.0378	0.0481	0.786	0.4346				
breakpoint: AGE = 38)	β2	-0.1301	0.0603	-2.158	0.0342	*			
PPT_2 ~ AGE	Intercept	8.1804	1.6040	5.100	<0.0001	***	0.3834	F (2;74) = 24.63	<0.0001***
(piecewise linear<br>regression,	β1	0.1113	0.0554	2.011	0.0480	*			
breakpoint: AGE = 32)	β2	-0.1895	0.0619	-3.060	0.0031	**			
PPT_A ~ AGE	Intercept	8.5186	1.0547	8.077	<0.0001	***	0.5741	F (2;74) = 52.22	<0.0001***
(quadratic regression)	β1	0.0593	0.0462	1.285	0.2029				
	β2	-0.0013	0.0005	-2.862	0.0055	**			
Grip Force ~ AGE	Intercept	23.1250	9.1146	2.537	0.0133	*	0.1318	F (2;74) = 6.77	0.0020**
(piecewise linear<br>regression,	β1	0.5566	0.2754	2.021	0.0469	*			
breakpoint: AGE = 39)	β2	-0.9818	0.3539	-2.774	0.0070	**			
Pinch Force ~ AGE	Intercept	5.0389	0.4589	10.981	<0.0001	***	0.0549	F (1;75) = 5.415	0.0227*
(linear regression)	β1	-0.0203	0.0087	-2.327	0.0227	*			
$AREA_{FDI}$ ~ AGE	Intercept	16.5760	1.6883	9.818	<0.0001	***	0.0009	F (1;74) = 1.069	0.3045
(linear regression)	β1	-0.0332	0.0321	-1.034	0.3050				
$ln(VOL_{FDI})$ ~ AGE	Intercept	3.9738	0.5325	7.462	<0.0001	***	0.1793	F (2;71) = 8.976	0.0003***
(piecewise linear<br>regression,	β1	-0.0535	0.0143	-3.742	0.0004	***			
Breakpoint: AGE = 46 ‡)	β2	0.0600	0.0217	2.762	0.0073	**			
$\sqrt[3]{MAXMEP_{FDI}}$ ~ AGE	Intercept	1.8830	0.1766	10.664	<0.0001	***	0.1893	F (2;71) = 9.521	0.0002***
(piecewise linear<br>regression,	β1	-0.0192	0.0047	-4.056	0.0001	***			
breakpoint: AGE = 46 ‡)	β2	0.0230	0.0072	3.192	0.0021	**			
$AREA_{ADM}$ ~ AGE	Intercept	11.7541	2.0456	5.746	<0.0001	***	-0.0154	F (1;65) = 0.0001	0.9940
(linear regression)	β1	-0.0003	0.0385	-0.008	0.9940				
VOL_{ADM} ~ AGE	Intercept	3.4512	1.1072	3.117	0.0027	**	-0.0148	F (1;65) = 0.0407	0.8408
(linear regression)	β1	-0.0042	0.0208	-0.202	0.8408				
$MAXMEP_{ADM}$ ~ AGE	Intercept	0.9189	0.2718	3.381	0.0012	**	-0.0117	F (1;65) = 0.2360	0.6287
(linear regression)	β1	-0.0025	0.0051	-0.486	0.6287				

The following regression models were used: linear regression $\hat{Y} = \beta_0 + \beta_1 X$; quadratic regression $\hat{Y} = \beta_0 + \beta_1 X + \beta_2 X^2$;

piecewise linear regression $\hat{Y} = \beta_0 + \beta_1 X + \beta_2 (X - X_{BP}) \times D \; with \; D = \begin{cases} 0 \; for \; X \le X_{BP} \\ 1 \; for \; X > X_{BP} \end{cases}$ (BP = breakpoint).

(‡ = two influential data points [aged 18 and 19 years] removed in order to fulfill model assumptions; significant p-values are indicated with asterisks (*** p < 0.001; ** p < 0.01; * p < 0.05) and printed in bold)

Abbreviations: ADM = abductor digiti minimi muscle; AREA = area of cortical motor representation; DEN = denominator; DF = degrees of freedom; FDI = first dorsal interosseus muscle; MAXMEP = maximal motor evoked potential of cortical motor representation; NUM = numerator; PPT_1/_2/_A = Purdue Pegboard Test unimanual/bimanual/assembly subtest; SE = standard error; VOL = volume of cortical motor representation.
